# Bio‐inspired optical structures for enhancing luminescence

**DOI:** 10.1002/EXP.20220052

**Published:** 2023-04-11

**Authors:** Zemin Zhang, Florian Vogelbacher, Yanlin Song, Yang Tian, Mingzhu Li

**Affiliations:** ^1^ Key Laboratory of Green Printing, Institute of Chemistry Chinese Academy of Sciences Beijing P. R. China; ^2^ Beijing Key Laboratory for Optical Materials and Photonic Devices, Department of Chemistry, Beijing Advanced Innovation Center for Imaging Technology Capital Normal University Beijing P. R. China; ^3^ Key Laboratory of Materials Processing and Mold of Ministry of Education Zhengzhou University Zhengzhou P. R. China

**Keywords:** anti‐counterfeiting, bio‐inspired optical structure, light‐emitting source, luminescence enhancement, sensing

## Abstract

Luminescence is an essential signal for many plants, insects, and marine organisms to attract the opposite sex, avoid predators, and so on. Most luminescent living organisms have ingenious optical structures which can help them get high luminescent performances. These remarkable and efficient structures have been formed by natural selection from long‐time evolution. Researchers keenly observed the enhanced luminescence phenomena and studied how these phenomena happen in order to learn the characteristics of bio‐photonics. In this review, we summarize the optical structures for enhancing luminescence and their applications. The structures are classified according to their different functions. We focus on how researchers use these biological inspirations to enhance different luminescence processes, such as chemiluminescence (CL), photoluminescence (PL), and electroluminescence (EL). It lays a foundation for further research on the applications of luminescence enhancement. Furthermore, we give examples of luminescence enhancement by bio‐inspired structures in information encryption, biochemical detection, and light sources. These examples show that it is possible to use bio‐inspired optical structures to solve complex problems in optical applications. Our work will provide guidance for research on biomimetic optics, micro‐ and nano‐optical structures, and enhanced luminescence.

## Graphical abstract

Many organisms had formed ingenious optical structures for the efficient emission of light signals in the environment during the long‐term evolution. We summarized detailed examples of bio‐inspired optical structures in enhanced luminescence and presented the applications of these inspirations in information encryption, biochemical detection, and luminescent sources. Our work will provide profound guidance for bio‐inspired optical structures in bio‐photonics, nano‐photonics, and other fields.

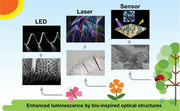

## 1 INTRODUCTION

Luminescent materials are widely applied in the fields of sensors, information anti‐counterfeiting, light‐emitting diodes (LEDs), and laser light sources. Due to the limitation of the material properties, the luminescent performance of a device needs to be improved to satisfy the strict requirements of practical applications. For example, fluorescent anti‐counterfeiting applications require high security, multiple information encryption, and simple operation.^[^ ^1^, ^2^, ^3^, ^4^ ^]^ Detection devices based on luminescent emission depend on an inexpensive miniaturized system design, high reliability, and fast response.^[^ ^5^, ^6^, ^7^ ^]^ In lighting applications, high light extraction efficiency, high luminous brightness, and controllable emission directivity are required.^[^ ^8^, ^9^, ^10^, ^11^ ^]^

Recently, bio‐inspired optics have received extensive attention and provide ingenious ideas for researchers.^[^ ^12^, ^13^, ^14^, ^15^, ^16^ ^]^ Many organisms are able to emit light to attract mates or intimidate enemies via the processes of bioluminescence, fluorescence, or phosphorescence. They evolved many unique optical structures, which produce wonderful effects of enhancing light extraction, making luminescence directional, and so on.^[^ ^17^, ^18^, ^19^, ^20^ ^]^ Researchers found inspiration from them and exploited these optical phenomena to develop photonic devices.^[^ ^21^, ^22^, ^23^, ^24^, ^25^ ^]^ With the development of nanomaterials and nanotechnology, many processing techniques were used to fabricate different bio‐inspired optical structures.^[^ ^26^, ^27^, ^28^ ^]^ The manufacturing methods of structured luminescent devices include depositing luminescent nanomaterials on biological templates as well as using imprinting or photolithography to introduce optical structures to the luminescent materials.^[^ ^29^, ^30^, ^31^ ^]^ Furthermore, calculations and simulation results of bio‐inspired optical structures help people to understand the fundamentals of these optical structures and guide the design and fabrication of luminescent devices. The smart solutions in organisms to address optical challenges provide plenty of motivations for developing novel artificial photonic materials and luminescent devices, such as color filters, artificial eyes, and electronic skin.^[^ ^32^, ^33^, ^34^ ^]^

**FIGURE 1:**
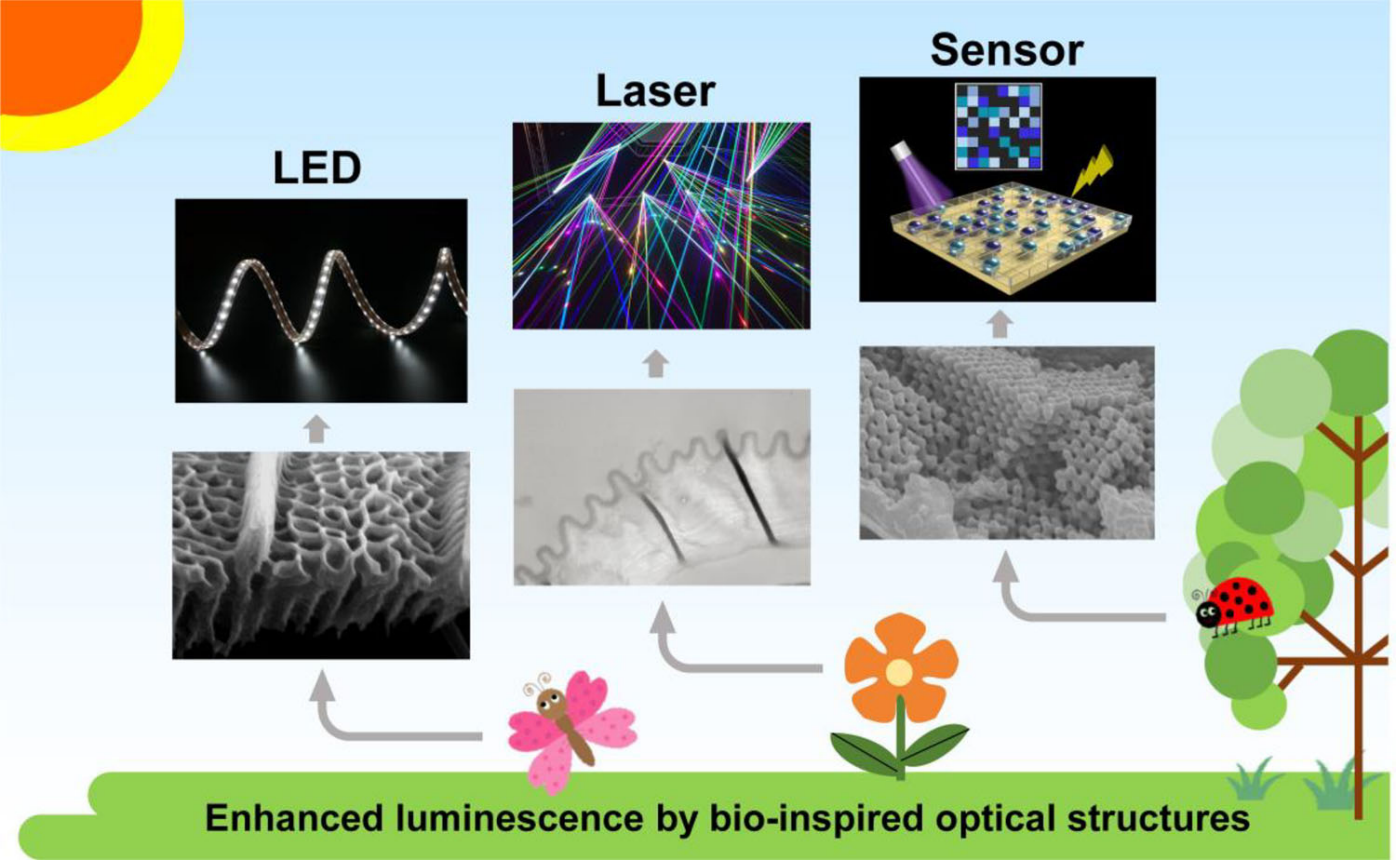
Schematic illustration of optical structures for enhancing luminescence observed from organisms and their applications in LED, lasers, and sensors. Reproduced with permission.^[^ ^20^ ^]^ Copyright 2017, Springer Nature; Reproduced with permission.^[^ ^35^ ^]^ Copyright 2007, American Physical Society; Reproduced with permission.^[^ ^42^ ^]^ Copyright 2005, AAAS.

Here, we focus on enhancing luminescence by bio‐inspired optical structures and their application to luminescent devices (Figure 1). First, we classify the organisms with actively or passively optical emitting signals as well as their micro‐ and nano‐ optical structures. Second, we report bio‐inspired optical structures to enhance different types of luminescence processes and their applications in anti‐counterfeiting, biochemical detection, and light sources. Finally, we summarize advantages, disadvantages, and improvement methods of bio‐inspired optical structures for enhancing luminescence.

## 2 OPTICAL STRUCTURES IN ORGANISMS FOR ENHANCING OPTICAL SIGNALS

Luminescence exists in a series of organisms, such as marine organisms, plants, and insects.^[^ ^35^, ^36^, ^37^, ^38^ ^]^ Analysis and interpretation of these fluorescent signals can provide information about their physiological state, how species communicate with each other, and the presence of specific chemicals. Many interesting interactions occur between optical structures and luminescence produced by pigments. With the development of microscopic technology and the improved accuracy of observation, researchers have revealed the optical structures existing on surfaces of organisms and studied them in detail.^[^ ^39^, ^40^ ^]^

### 2.1 Enhanced fluorescence emission

**FIGURE 2:**
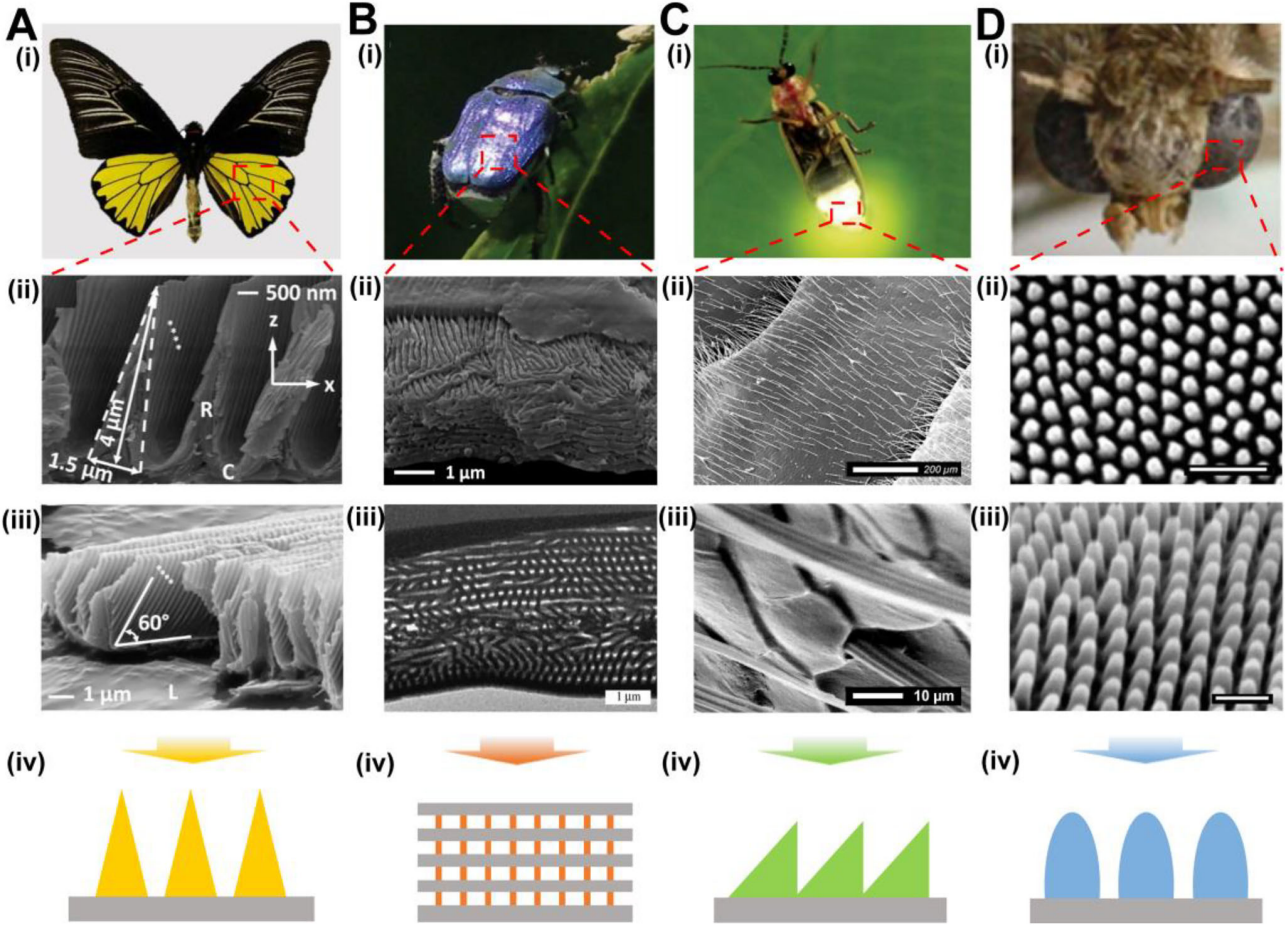
(A) The hindwings of *Troïdes magellanus* butterflies exhibit a uniform yellow coloration under daylight and enhanced yellow‐green coloration under ultraviolet illumination. The scanning electron microscope (SEM) images of cross and lateral views show that ridge structures with triangular cross‐sections are arranged as a grating structure, and each ridge has a set of lamellae. Reproduced with permission.^[^ ^43^ ^]^ Copyright 2012, Optical Society of America. (B) The *Hoplia coerulea* beetle displays a violet‐blue coloration. The scales covering the elytra consist of alternating films of pure and porous mixed air‐cuticle layers forming a periodic photonic structure. Reproduced with permission.^[^ ^45^ ^]^ Copyright 2016, The Royal Society. (C) Fireflies can emit bright light from their abdominal segments with complex optical structures. The mismatched scales on the ventral side result in an enhanced emission extraction. Reproduced with permission.^[^ ^49^ ^]^ Copyright 2013, Optical Society of America. (D) The corneal surface of moth eyes is lined with nanopillar structures. The structures with anti‐reflection effects increase the input of light. Reproduced with permission.^[^ ^51^ ^]^ Copyright 2014, Wiley‐VCH.

In 2005, researchers discovered an internal light‐filtering effect in the flowers of *Mirabilis jalapa*. There are two pigments (betaxanthin and betacyanin) in flowers for generating fluorescence, and the emission of betaxanthin is absorbed by betacyanins. This special mechanism creates a showy pattern with contrasting fluorescent on the petals to enhance visibility to pollinators.^[^ ^41^ ^]^ This example demonstrates the important role of the enhanced fluorescence signal for the organism. Fluorescent pigments are also present in different photonic structures, which strongly control the intensity and direction of the emitted light. The swallowtail (*Papilio*) butterfly has pigment‐infused two‐dimensional (2D) photonic crystal (PhC) structures on its wing scales from colored regions. This distributed Bragg reflector (DBR) structure inhibits the in‐plane emission and enhances its out‐of‐plane emission, leading to directional fluorescence.^[^ ^42^ ^]^ The same phenomenon of directed fluorescence enhancement occurs in male butterflies *Troïdes magellanus* (*Papilionidae*) (Figure 2A) because the fluorophores in the hindwings are confined in the three‐dimensional (3D) structures consisting of periodically‐distributed ridges with triangular cross sections.^[^ ^43^ ^]^ The 3D structure serves as a diffraction grating that reflects light and couples fluorescence to a structure acting as an optical waveguide, leading to a directional emission. On the back of some beetles, natural pigments exist in the photonic structures of the elytra scales. For example, the male *Hoplia coerulea* beetle has a porous photonic structure that is embedded with fluorophores. This structure consists of thin pure cuticle layers with periodically arranged and mixed air‐cuticle porous layers (Figure 2B).^[^ ^44^ ^]^ The fluorophores in the structures show, depending on the position of the photonic bandgap, enhanced or suppressed fluorescence emission because resonant structures can modify the density of optical states (DOS), which is related to electromagnetic modes of propagation, the decay rate of fluorescence, and the lifetime of the excited state.^[^ ^45^ ^]^

### 2.2 Enhanced light extraction

Some organisms can generate bioluminescence through a chemical reaction in their bodies.^[^ ^46^, ^47^, ^48^ ^]^ However, the refractive index mismatch at interfaces always causes reflection and loss of light. Surprisingly, organisms have evolved special optical structures which can extract light from the high‐refractive‐index interior to the air. For example, fireflies can efficiently output light for intimidating enemies or attracting a mate. Bay and co‐workers studied multi‐dimensional optical structures existing in the luminous area of the firefly's body (Figure 2C).^[^ ^49^ ^]^ The edge structures from jagged and misfitted scales lead to enhanced light extraction by diffuse transmission. The reduction of the refractive index in the emission region also contributes to the efficient extraction of the emission. The nipple array structures on the moth‐eye surface form anti‐reflection structures which reduce the glare of the eyes and avoid detection by predators.^[^ ^50^ ^]^ Oh and co‐workers reported that the nanopillar array structures with a ∼0.5 fill factor, that is, the relative areal fraction of sub‐wavelength nanostructures, achieved a gradual decrease in reflectivity with increasing height (Figure 2D). The structures can be used to increase light transmittance and improve the sensitivity of moth eyes to sunlight.^[^ ^51^ ^]^ The same phenomenon has been found on the wings of butterflies. For example, the randomly‐distributed height of pillar structures with a mean height of 500 nm and a variance of 100 nm on the wings of glasswing butterflies leads to low broadband reflection below 2.2% for normal incidence and large view angles.^[^ ^52^ ^]^ Fabricating the anti‐reflection structures can effectively extract emission light, resulting in the enhanced luminescence performance of light‐emitting devices.

## 3 BIO‐INSPIRED OPTICAL STRUCTURES FOR HIGHLY EFFICIENT LUMINESCENCE

As we saw in the previous section, in order to adapt to changes in the environment and ensure survival, organisms have already created some fantastic optical structures. Therefore, studying the underlying mechanism in the structures for enhancing luminescence plays an essential role in enabling practical applications of luminescent materials. Based on the source of excitation, luminescence can be classified into different types, such as chemiluminescence (CL), photoluminescence (PL), and electroluminescence (EL). Luminescent materials that are used in CL, PL, and EL processes mainly include rare‐earth luminescent materials, quantum dot luminescent materials, and organic luminescent materials. In addition to selecting luminescent materials with matching energy levels, efficient carrier transport, and high luminous efficiency, it is also necessary to match the size of the optical structure to the emission wavelength to achieve the highest luminescence enhancement. Researchers made many efforts to use bio‐inspired optical structures to enhance different luminescence types, which are described in detail in the following subsections.

### 3.1 Enhanced CL

**FIGURE 3:**
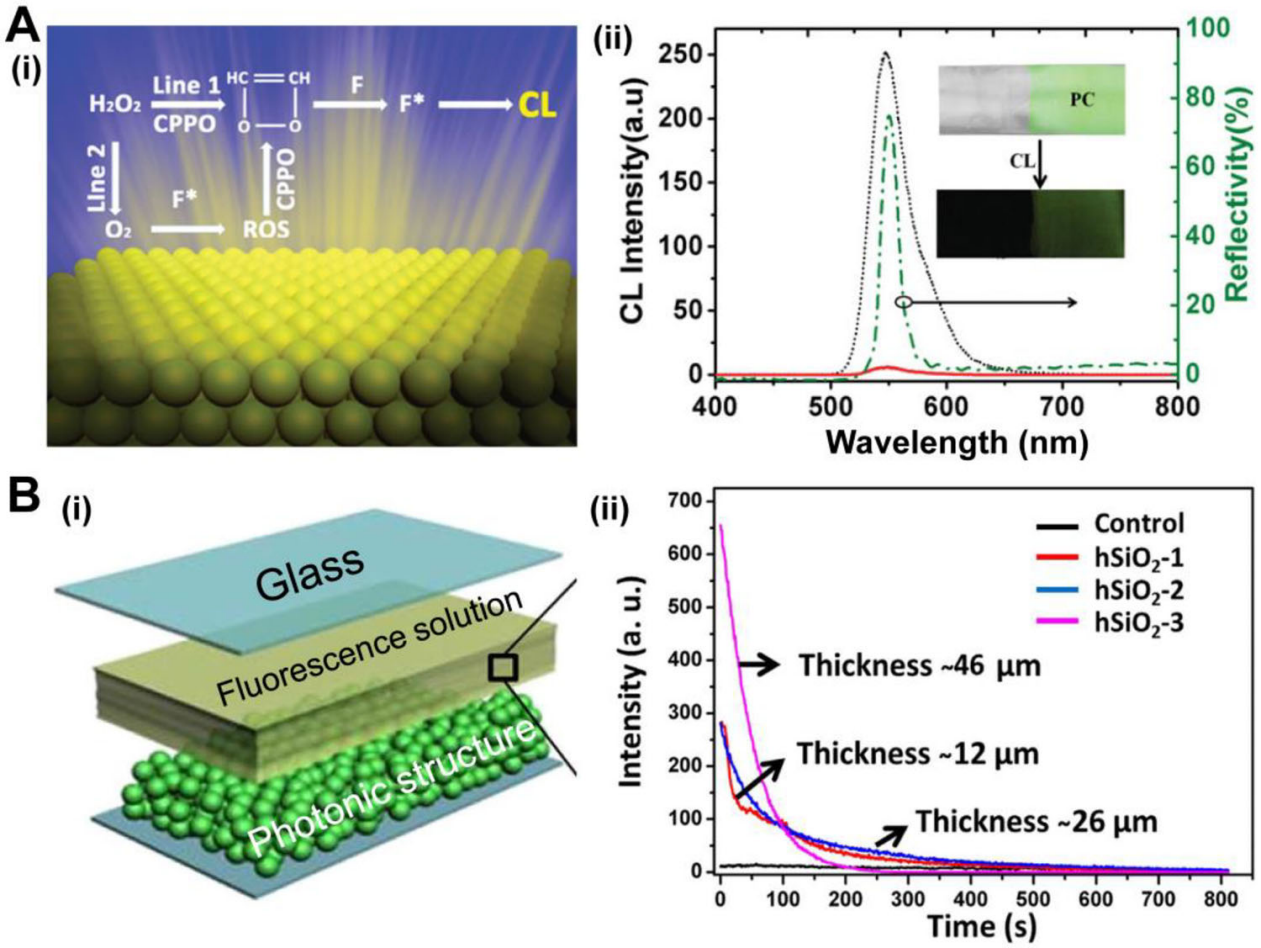
Bio‐inspired optical structures enhance chemiluminescence. (A) (i) Illustration of the mechanism for the PhC‐enhanced CL intensity and the decay rate of CL process. (ii) Short dot line and solid line are CL spectra of samples with and without structures, respectively. The dash‐dot line represents the reflection spectrum of the PhC structure. The inset pictures show the green PhC film on a glass substrate and the increasing CL intensity in the PhC area. Reproduced with permission.^[^ ^54^ ^]^ Copyright 2013, Wiley‐VCH. (B) (i) The composition of the bioinspired CL light‐emitted device. (ii) The CL intensity versus time for a control sample and experimental samples with different photonic structure thicknesses. Reproduced with permission.^[^ ^55^ ^]^ Copyright 2015, Springer Nature.

During chemical reactions, excited substances emit a type of light radiation called CL. CL can be used to determine reactants and catalysts in chemical reactions and has been developed for biochemical analysis and detection.^[^ ^53^ ^]^ It is produced on three conditions: i) the reaction must provide sufficient excitation energy, ii) the chemical energy can be accepted by the substance and generate an excited state, and iii) the excited state molecule must have high quantum efficiency to release photons. However, due to the low fluorescence quantum yield and weak luminescence intensity, it is difficult for CL systems to achieve the requirements of high‐sensitivity sensors for biochemical analysis and detection. Bio‐inspired optical structures offer inspiration to improve the quantum yield and luminescence intensity of CL. Shi and co‐workers proposed a strategy to enhance CL using PhC structures (Figure 3A).^[^ ^54^ ^]^ The 3D PhC structure suppresses the emission at the central wavelength of the stopband and enhances the emission at the edge of the stopband. The PhC structure with matched photonic stopband makes the CL achieve a 44.9‐times enhancement of emission intensity and the highest decay rate. Meanwhile, the advantages of this large‐area PhC film are the decreased consumption of excitons and the weak quenching of fluorescence. In addition, researchers became interested in the CL system in fireflies. Chen and co‐workers analyzed the optical structures in the reflective layer of firefly lanterns and fabricated an artificial film with high reflectivity for enhancing CL (Figure 3B).^[^ ^55^ ^]^ They combined a chemical solution and randomly distributed hollow silicon particles between two glasses. This photonic glass exhibited high reflectivity over a broad wavelength region, facilitating CL extraction. The hollow particles with high specific surface enhanced transfer of the interface and increased the rate of reaction, leading to a 55.3‐times improved CL intensity. This bio‐inspired investigation provided a new avenue to design the structures for enhancing CL, which will be beneficial in sensors, imaging, and light sources.

### 3.2 Enhanced PL

PL is the process of a fluorophore undergoing a radiative transition from an excited state back to its ground state and generating photons. The different photoluminescent materials have different positions of the localized energy levels in the forbidden band leading to distinct radiative transitions. PL materials include long afterglow materials, up‐conversion materials, aggregation‐induced emission (AIE) materials, and so on.^[^ ^56^, ^57^, ^58^, ^59^, ^60^, ^61^, ^62^ ^]^ The environments also affect the spontaneous emission of luminescent materials in addition to the intrinsic electronic properties of the material. For example, nanoscale resonant cavities can enhance the spontaneous emission rate of luminescent materials based on the Purcell effect. The enhancement or suppression of spontaneous emission can be realized by changing the density of states of the electromagnetic field through optical structures. To improve the quantum efficiency and luminous intensity of PL, it is necessary to combine luminescent materials with optical structures.

**FIGURE 4:**
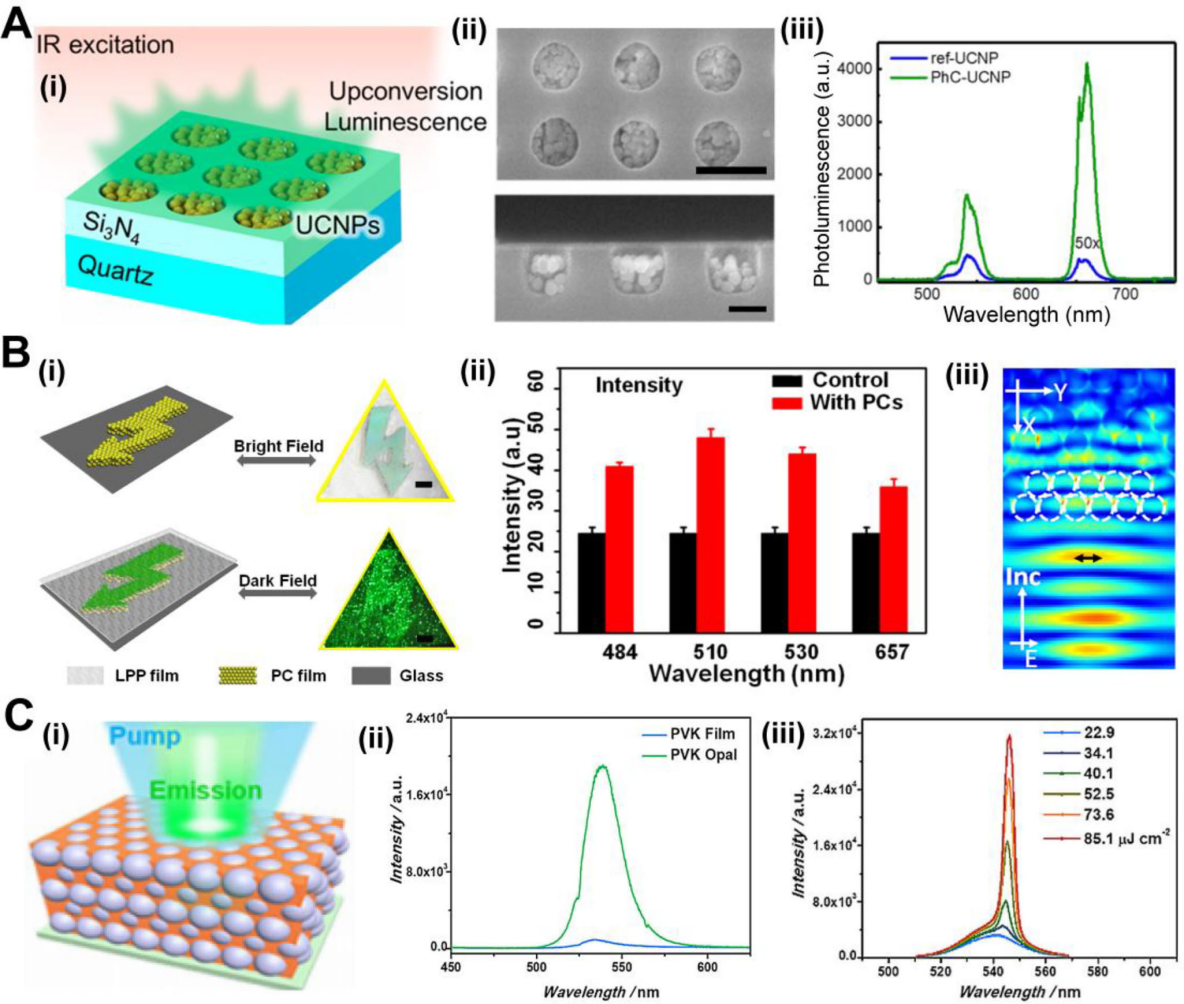
Bio‐inspired optical structures enhance photoluminescence. (A) (i) The schematic of the enhanced upconversion luminescence by two‐dimensional PhC patterns. (ii) The scanning electron microscope (SEM) images in top‐down and cross‐sectional views of UCNP‐filled PhC film sample, scale bars are 500 nm (upper) and 200 nm (bottom). (iii) The PL intensity spectra of the control sample and PhC‐UCNP sample under the excitation of near infrared (NIR) lasing. Reproduced with permission.^[^ ^64^ ^]^ Copyright 2019, American Chemical Society. (B) (i) The fabrication of PhC structured film with a warning pattern. The schematic of enhanced long persistent phosphor luminescence emitted from the device. (ii) The comparison of afterglow intensities of SrAl_2_O_4_: Eu material with and without wavelength‐tunable PhC. (iii) The numerically calculated E‐field inside the PhC structure irradiated by a Gaussian beam. Reproduced with permission.^[^ ^66^ ^]^ Copyright 2014, American Chemical Society. (C) (i) The diagram of enhanced amplified spontaneous emission from a halide perovskite opal sample under the pump lasing. (ii) Fluorescence spectra of the perovskite film with (green) and without (blue) the opal structure. (iii) The intensity spectra of emission from the perovskite opal film with the increasing pump power. Reproduced with permission.^[^ ^68^ ^]^ Copyright 2018, Wiley‐VCH.

Upconversion nanoparticles (UCNPs), which can convert near‐infrared light to visible light, become possible through two‐photon or multi‐photon processes. To improve the luminescence intensity and energy conversion efficiency, Yin and co‐workers presented a significantly increased upconversion luminescence by combining the band edge effect of 3D opal PhC and the surface plasmon effect of gold nanorods. The metal nanoparticles distributed on the luminescent films absorb the matched incident light and generate a localized surface plasmon resonance (LSPR) which transfers the energy to the material to control the luminescent properties. Numerical simulations of the electromagnetic field of the plasmonic gold nanorods showed a size‐dependent field enhancement. At the same time, it was observed that small UCNPs lead to luminescent centers within the interaction range of the gold nanorods. When the average size of the UCNPs is 5 nm, the length of the gold nanorod is 115 nm, and the photonic stop band (PSB) of an opal PhC structure is at 980 nm, the upconversion luminescence of UCNPs had been enhanced by more than 10^3^ folds, while the detectable excitation threshold had been reduced by three orders of magnitude to 0.37 mW mm^−2^.^[^ ^63^ ^]^ Mao and co‐workers incorporated UCNPs into a 2D PhC film by electrostatic interactions on the surface. The discontinuous dielectric PhC structure film forms a strongly enhanced local electric field region, which resulted in approximately 130‐ and 350‐fold enhancements for green and red luminescence, respectively (Figure 4A).^[^ ^64^ ^]^ The 2D PhC film can be applied to large‐area devices. Gao and co‐workers employed butterfly wings with PhC structures as a biological template to improve the luminescence of UCNPs. They achieved enhanced luminescence by tuning the bandgaps of PhC structures. The results showed that the increased local DOS led to the enhancement of decay rates. Overall, the hybrid PhC structures are suitable for producing biophotonic devices with tunable upconversion emission.^[^ ^65^ ^]^ The PhC structure can also be used for long afterglow materials to enhance the emission intensity and prolong the glowing time. Shi and co‐workers assembled PhC on a substrate and covered them with a mixture of SrAl_2_O_4_:Eu and polydimethylsiloxane (PDMS) (Figure 4B).^[^ ^66^ ^]^ Due to the bandgap of PhC structures, the luminescence intensity and the emission direction are improved. The reflected light is reabsorbed and stimulates the luminescence. With an optimized lattice constant of the PhC structure, a two‐times afterglow intensity and a 1.7‐times afterglow time of SrAl_2_O_4_:Eu without any dopants were achieved. Enhancing the luminescence of afterglow materials with special light storage and release properties is of great interest for displays, lighting, and security signals.

Perovskite materials with high fluorescence quantum yield and tunable wavelength are widely applied in light‐emitting devices such as LEDs and lasers. Therefore, the enhancement of luminescence becomes essential for the applications of perovskite emitters. Suppression and redistribution of spontaneous emission caused by PhC structures occur in grating and opal patterned perovskite devices. Wang and co‐workers introduced a subwavelength grating metasurface structure to enhance the PL of perovskite films by nanoimprinting technique.^[^ ^67^ ^]^ The simulation results of light transport spectroscopy indicated the existence of high‐quality cavity modes. Steady‐state PL spectra showed that the metasurface structure significantly enhances the PL intensity by a factor of eight because the optimization of the metasurface leads to great dielectric resonance. Zhou and co‐workers used the opal template to prepare 3D perovskite PhC (Figure 4C).^[^ ^68^ ^]^ Amplified spontaneous emission of the 3D perovskite PhC appeared at a low‐power threshold under lasing excitation because of the strong coherent scattering and a high‐intensity resonant field of the PhC structure. Furthermore, the PhC structures also can control the propagation of emission light and improve the in‐coupling and out‐coupling of radiation by matching resonant modes of PhC and both the excitation and the emission of the materials.^[^ ^69^ ^]^ The perovskite nanocrystals on the top of PhC structures achieved a sixteen times enhancement of the PL intensity and an increased spontaneous emission rate.

### 3.3 Enhanced light extraction for highly efficient EL

**FIGURE 5:**
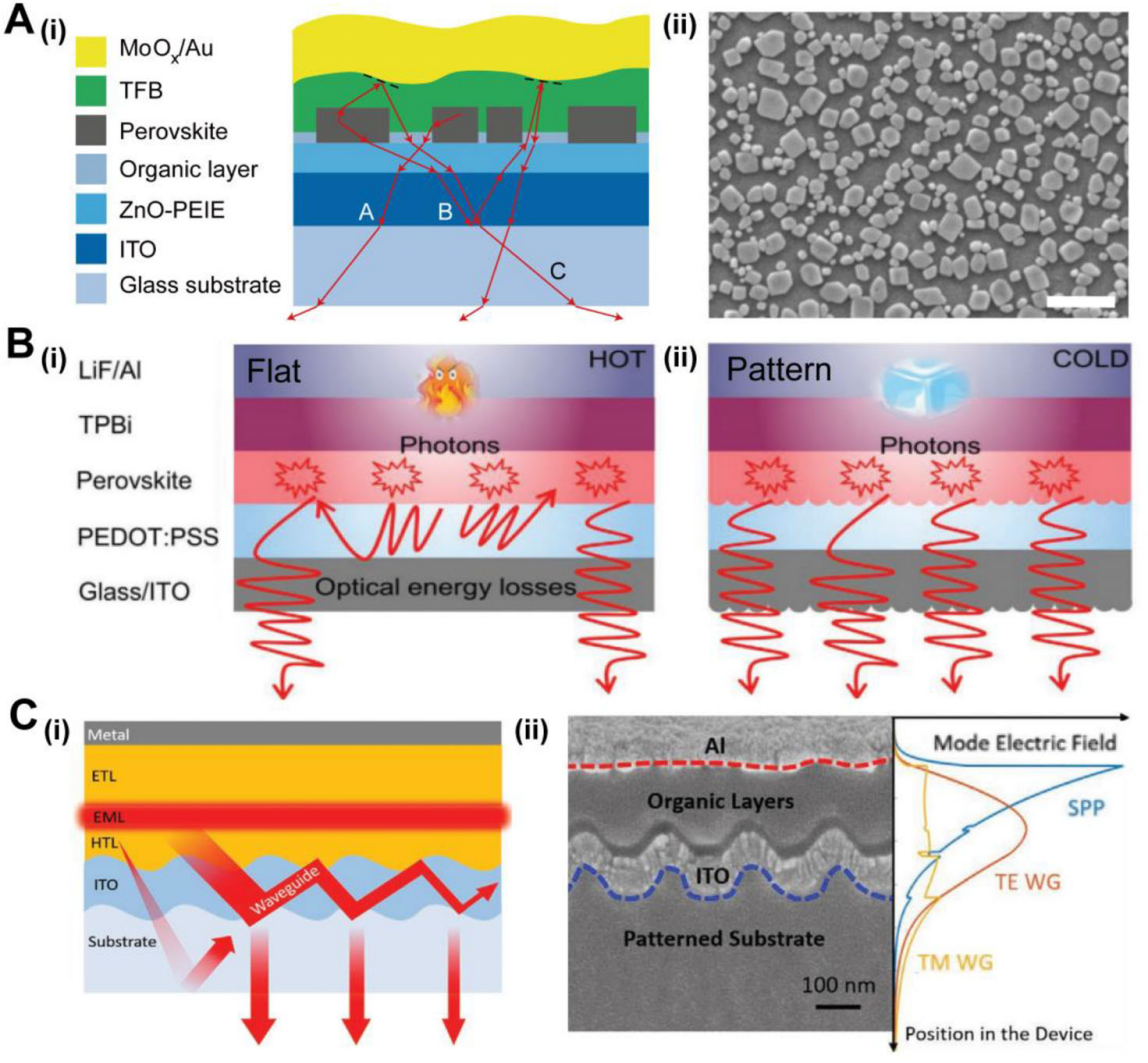
Bio‐inspired optical structures enhance electroluminescence. (A) (i) The schematic shows the layers of the perovskite LED device. The submicrometre structure extracts the trapped light (red rays). (ii) The SEM image of perovskite film (scale bar is 1 μm). Reproduced with permission.^[^ ^72^ ^]^ Copyright 2018, Springer Nature. (B) Schematic illustration of the performance improvement mechanism and photon trajectories for (i) flat and (ii) patterned perovskite LEDs. The patterned structures can decrease the optical energy losses and reduce the heating of the devices. Reproduced with permission.^[^ ^73^ ^]^ Copyright 2021, Wiley‐VCH. (C) (i) The schematic of the LED device with directional emission by enhancing the extraction of the waveguide mode. (ii) Cross‐sectional SEM image of the grating structured LED device. The finite‐difference time‐domain simulation results show the electric field intensity (|E|^2^) distribution of modes in a LED. Reproduced with permission.^[^ ^74^ ^]^ Copyright 2021, Wiley‐VCH.

In the EL process, photons are generated due to the injection of an applied current into the layer containing the active material. Various semiconductor materials with high PL quantum efficiency, such as GaN, organic molecule, perovskite, have been employed to realize EL processes. However, the external quantum efficiency (EQE) of EL is still low due to the limitations originating from non‐radiative recombination and light trapping from defects.^[^ ^70^, ^71^ ^]^ Cao and co‐workers introduced additives into a precursor solution to prepare perovskite thin films with submicrometre platelets (Figure 5A).^[^ ^72^ ^]^ These randomly distributed submicrometre structures enhanced the extraction of all‐direction light without spectrum shift and angular dependence. Furthermore, the amino acids passivate the defects in the perovskite surface, leading to reducing the non‐radiative recombination. This LED achieved 20.7% EQE and 12% energy conversion efficiency. Ye and co‐workers combined a perovskite active layer with an improved light outcoupling structure (Figure 5B).^[^ ^73^ ^]^ This photonic structure enhanced the extraction of light from trapped modes and reduced emission losses caused by the reabsorption in the perovskite layer, which maintained a low temperature in the device after prolonged operation. Finally, a long‐lived red LED with an EQE of 21.2% was realized. To achieve LEDs with directional emission and polarization characteristics, challenges concerning the characteristics of traditional Lambertian light sources need to be solved. Fu and co‐workers used a grating as a DBR to suppress air modes and extract waveguide modes for shaping beams (Figure 5C).^[^ ^74^ ^]^ The patterned samples only had strong diffraction peaks from the transverse electric (TE) waveguide mode, which corresponded to high directivity with a small divergence angle. The LEDs with highly directional beam shapes have great potential in solid‐state lighting, holographic displays, and stereoscopic displays.

## 4 APPLICATIONS OF ENHANCED LUMINESCENCE

### 4.1 Information encryption technology

**FIGURE 6:**
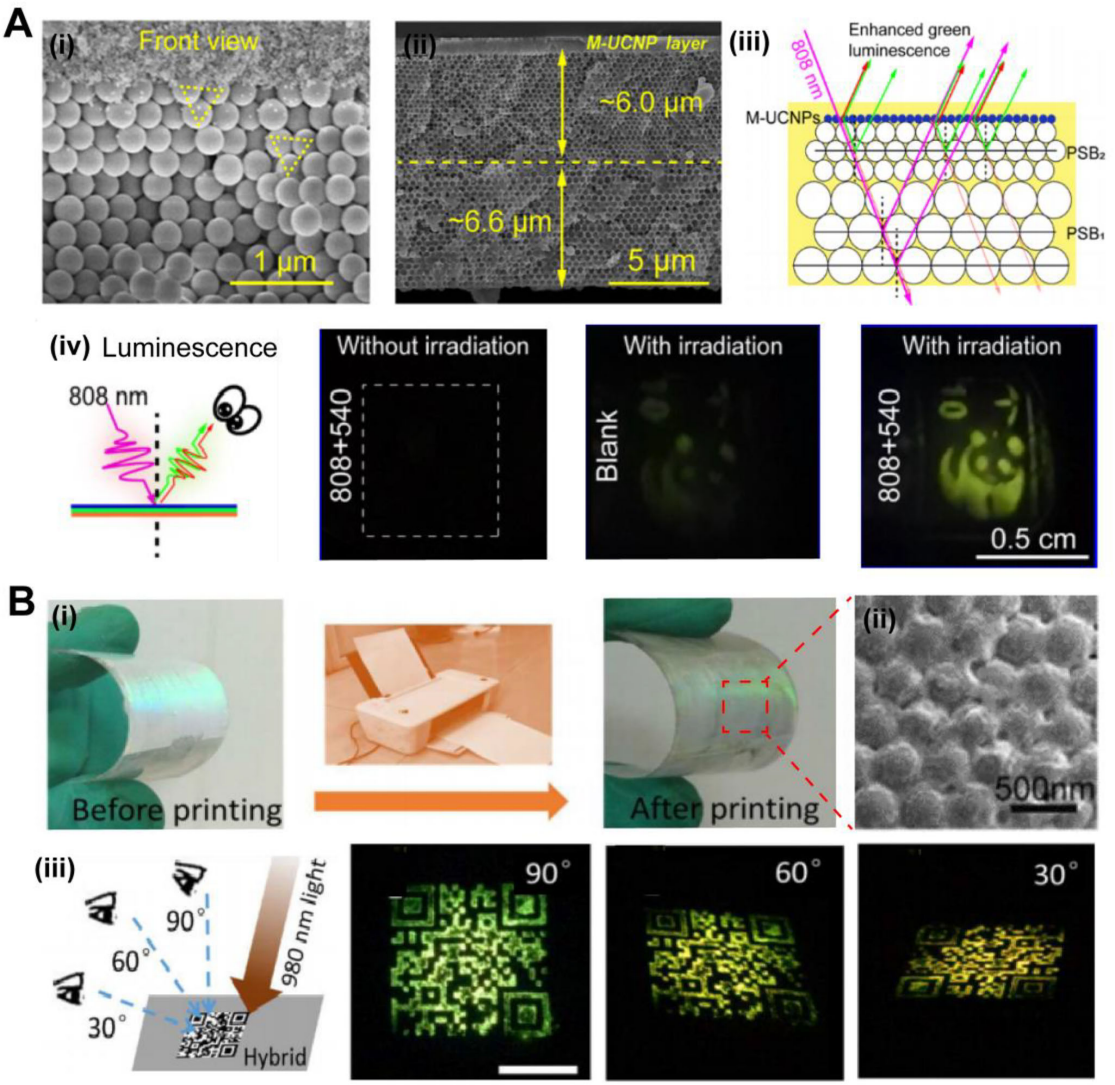
The application of enhanced luminescence in information encryption. (A) (i) The cross‐sectional SEM micrograph of bilayer structure consisted of the opal PhC (OPC) and hydrophilic modified UCNPs (M‐UCNPs). (ii) Cross‐sectional SEM micrograph of the inverse opal PhCs (IOPCs) and M‐UCNPs bilayer structure. (iii) The IOPC/M‐UCNP structure with double PSBs shows the enhanced luminescence. (iv) Schematic diagram for the observation of luminous patterns and digital photos of the blank sample and the “808+540” structure with and without irradiation of the laser. Reproduced with permission.^[^ ^78^ ^]^ Copyright 2022, American Chemical Society. (B) (i) Schematic of imprinting hybrid nanoparticles (Cu_2−x_S NPs and UCNPs) on the flexible film with OPC structures. (ii) SEM image of the hybrid nanoparticles sample. (iii) The anti‐counterfeiting hybrids sample shows angle‐dependent fluorescence. The photographs are taken at different angles under the NIR excitation. Reproduced with permission.^[^ ^79^ ^]^ Copyright 2017, American Chemical Society.

Information encryption technology is a protection strategy used to ensure the safe and effective transmission during an exchange of information. It is necessary to strengthen the security level of information and provide sufficient characters and encoding procedures in the communication industry.^[^ ^75^, ^76^, ^77^ ^]^ Many researchers exploited unique optical effects produced by photonic structures to enhance the luminescent signal or increase the number of information channels for highly‐secure information encryption. Meng and co‐workers designed a hydrophilic‐modified UCNPs integrated bilayer inverse opal PhC film.^[^ ^78^ ^]^ The schematic diagram in Figure 6A shows that the synergistic effect of the photonic bandgap of double layers realized optical amplification and enhanced the fluorescence signal. Furthermore, the researchers demonstrated the application of a triple anti‐counterfeiting technology based on the bilayer photonic structure and UCNPs. Under the irradiation of NIR lasing, the device exhibited a green luminescence pattern that cannot be seen under natural light. It ensured the anti‐counterfeiting ability and improved the reliability and security of information. Zhou and co‐workers demonstrated angle‐dependent anti‐counterfeiting applications based on synergistic upconversion enhancement caused by multiple physical effects (Figure 6B).^[^ ^79^ ^]^ They introduced flexible opal PhC employing a nanoimprinting technique. The printing pattern combined with UCNPs provided good optical contrast allowing high‐sensitivity detection. Because of the LSPR and two‐photon effect, angle‐dependent bright and clear quick response code patterns were seen under infrared laser excitation at different angles. This device can be applied to manufacturing flexible and large‐area security products. Physical unclonable function (PUF) provides another form of anti‐counterfeiting with advanced security. Wan and co‐workers replicated the micro‐ and nano‐structures on the surfaces of natural plants through a simple, green, and environmentally friendly fabrication process.^[^ ^80^ ^]^ The bio‐inspired PDMS films exhibited strong optical scattering effects under coherent illumination. This speckle response was used as a unidirectional and unique encoding mechanism. Compared with traditional digital cryptography, it was fundamentally secure because of the unreproducible structures from nature. The feasibility of using bio‐inspired PUFs as cryptographic primitives in information encryptions was experimentally validated, which showed the possible practical applications in verification and anti‐counterfeiting.

### 4.2 Highly sensitive sensors

**FIGURE 7:**
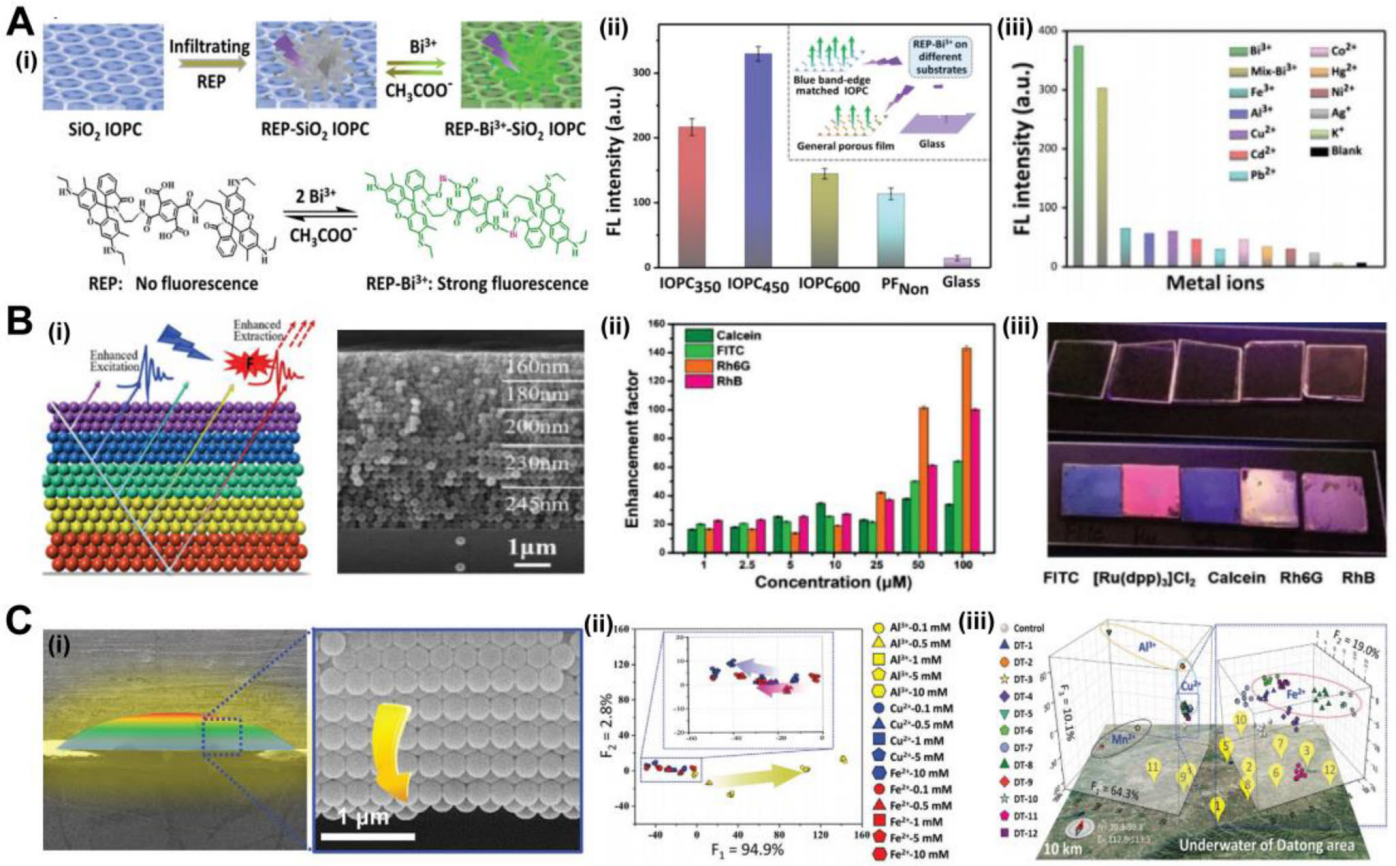
(A) (i) Schematic of fabricating a SiO_2_ IOPC film with enhanced fluorescence and chemical reaction between Rhodamine 6G derivative and Bi^3+^ ions. (ii) Fluorescence intensities of sensing samples with different surface topography tested in a Bi^3+^ solution. Inset: Schematic illustration of the enhanced fluorescence signal of the hybrid on the different substrates. (iii) The sensor shows different FL intensities for different metal ion solutions. Reproduced with permission.^[^ ^87^ ^]^ Copyright 2018, The Royal Society of Chemistry. (B) (i) Schematic of the combination of stopband effect and interference effect in the MHPhC structure (left) and the SEM image of the MHPhC samples (right). The different colors represent polystyrene spheres in different sizes. (ii) Enhancement factors of different fluorescent materials with increasing concentration on the MHPhC sample. (iii) The images under the UV lamp of different fluorescent materials covered on the glass and MHPhC sample. Reproduced with permission.^[^ ^88^ ^]^ Copyright 2018, Wiley‐VCH. (C) (i) SEM images of the 3D gradient colloidal PhC structure. (ii) The plot of scores of metal cations in gradient concentrations. The cluster distribution is scattered along the increased concentrations indicated by the arrows. (iii) The ion detection performance of the multimorphology PhC chip shows in the linear discriminant analysisscore plot which exhibits correct classification for all groundwater samples. Reproduced with permission.^[^ ^89^ ^]^ Copyright 2021, Wiley‐VCH.

Sensors need to detect extremely low concentrations of analytes in challenging physical and chemical backgrounds, which leads to expensive detection systems and complex multi‐step detection processes to achieve the required high sensitivity. To address these challenges, researchers enhanced the luminous signals by utilizing bio‐inspired optical structures.^[^ ^81^, ^82^, ^83^, ^84^ ^]^ For example, a common method for heavy metal ion detection is based on a fluorescent film sensor with immobilized sensitive molecules. The advantages are washability and reversibility of detection. The PhC structure with a photonic bandgap can be introduced to enhance the fluorescence signal for increased sensitivity.^[^ ^85^, ^86^ ^]^ Zhang and co‐workers constructed a fluorescence‐enhancing film sensor with a rapidly diffusible macroporous silica inverse opal structure (Figure 7A).^[^ ^87^ ^]^ The detection mechanism is that the reaction of a Rhodamine 6G derivative with Bi^3+^ ions emits an enhanced fluorescent signal. The intensity of fluorescence increased with increasing concentration. Moreover, the concentration of Bi^3+^ ions within a certain range can be quantitatively analyzed by a calibration curve. By adding different ions to the sensor, the stronger emission intensity resulted in a great selectivity for Bi^3+^ ions in the co‐detection of multiple ions. To further improve the detection limit of metal ions, Yan's group demonstrated multiple heterostructure PhC (MHPhC) films composed of continuous colloidal crystals with different diameters (Figure 7B).^[^ ^88^ ^]^ The wide bandgap of PhC structures enabled simultaneous matching of excitation and emission wavelengths, which enhanced the emission intensity of different dyes. To achieve successful multi‐recognition in large‐scale samples, sensors with diverse structures provide a possibility for sufficient information acquisition and signal enhancement. Huang and co‐workers designed PhC detection chips for multianalyte sensing with different micro‐ and nano‐structures by utilizing self‐assembled particles on hydrophilic patterns.^[^ ^89^ ^]^ Figure 7C shows the 3D morphology of the PhC in the array. The selective fluorescence enhancement caused by the periodic structure and observation angle lets the PhC distinguish the subtle differences in the response of different ions. The photonic bandgap was blue‐shifted from the center to the edge region of the PhC pixel. Experiments showed that the highly integrated and printable multianalyte chip with PhC structures achieved efficient detection of metal ions and was successfully applied to groundwater samples.

**FIGURE 8:**
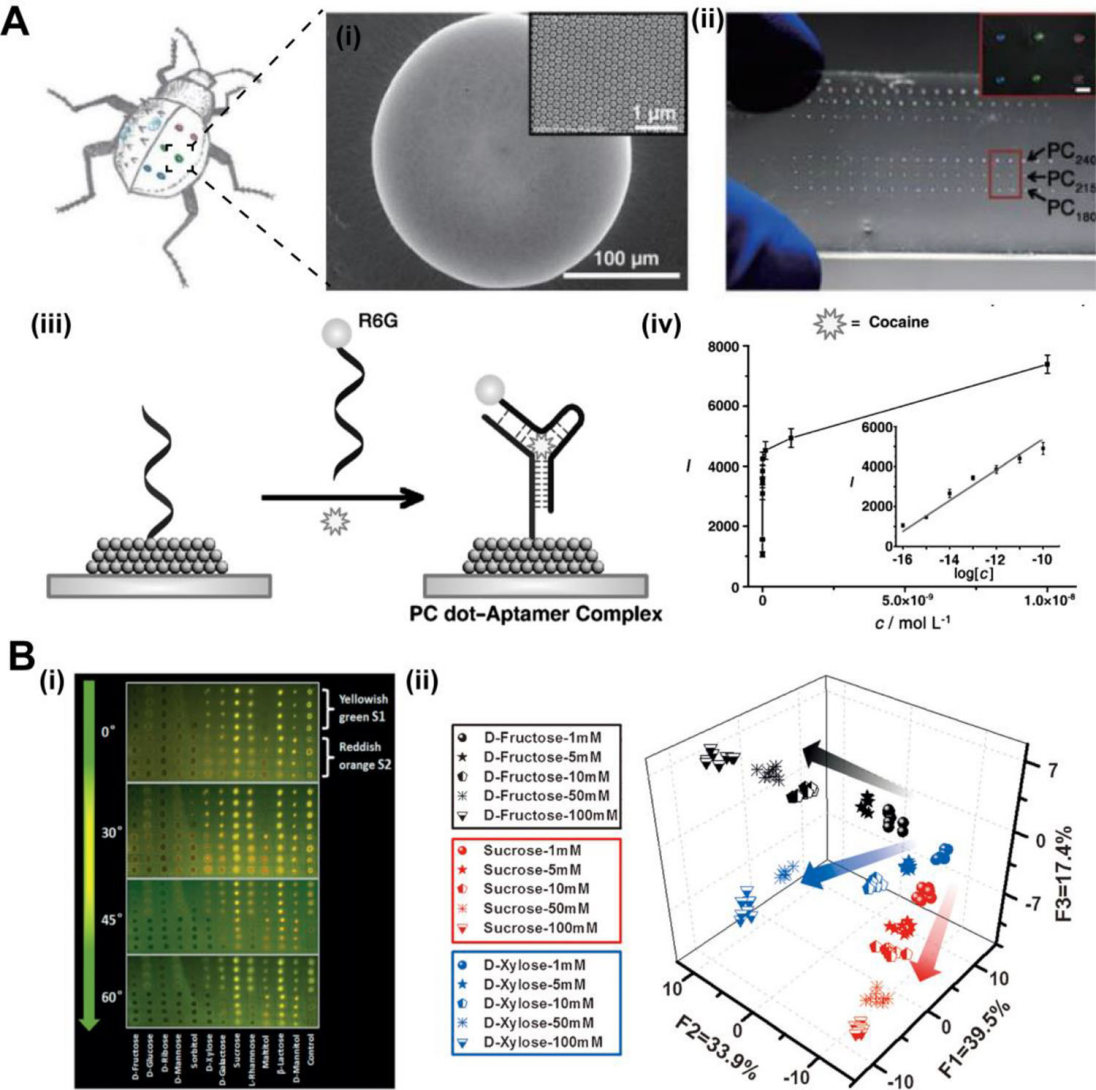
(A) The Stenocara beetle shows excellent water collecting ability due to hydrophilic and hydrophobic structures on the back. (i) SEM image shows a single PhC dot. The inset shows assembled colloidal spheres in the PhC dot. (ii) Photograph of the bio‐inspired PhC microchip with hydrophilic patterns on the hydrophobic substrate. The inset is the image of the PhC patterns with different stopbands. (iii) Schematic illustration of a DNA‐aptamer‐modified PhC microchip detecting cocaine. (iv) The intensities of fluorescence of cocaine with different concentrations. The fluorescence intensity and the logarithm of the cocaine concentration show a linear relationship. Reproduced with permission.^[^ ^94^ ^]^ Copyright 2014, Wiley‐VCH. (B) (i) The detection results of one control sample and twelve saccharides by the PhC chip. The different fluorescence signals detected at different angles. (ii) Plots of the scores of saccharides in gradient concentrations. The clusters are distributed along increasing concentrations indicated by arrows. Reproduced with permission.^[^ ^95^ ^]^ Copyright 2016, Wiley‐VCH.

**FIGURE 9:**
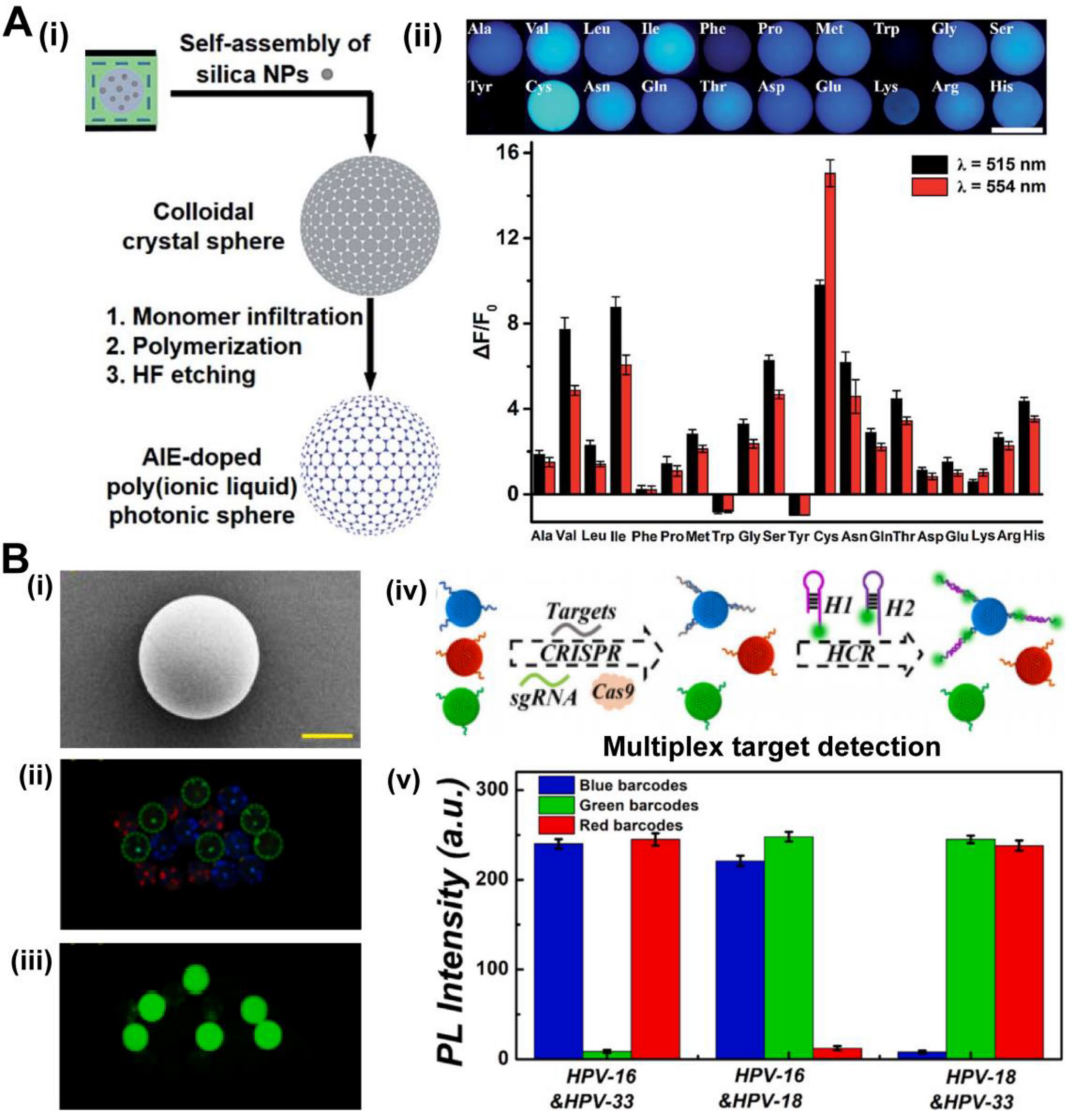
(A) (i) The fabrication of the AIE–doped poly(ionic liquid) photonic sphere. (ii) The photonic spheres exposed for 30 ms show bright fluorescence. Histogram of fluorescence enhancement in the sensing of 20 natural amino acids. Reproduced with permission.^[^ ^96^ ^]^ Copyright 2017, The Royal Society of Chemistry. (B) (i) SEM image of a single structural color microsphere. (ii) Optical and (iii) fluorescence microscopic images of PhC structured barcodes. (iv) Schematic diagram of the specific and multiplex target detection by polydopamine‐modified PhC microsphere. (v) The PL intensities of different PhC barcodes after being combined with two kinds of targets. Reproduced with permission.^[^ ^97^ ^]^ Copyright 2021, Elsevier.

Drug and biomolecule detection is widely used in early diagnosis and medical treatment.^[^ ^90^, ^91^, ^92^, ^93^ ^]^ However, detecting analytes from highly diluted solutions is still a challenge. Hou and co‐workers designed a PhC chip with hydrophilic and hydrophobic micropatterns inspired by desert beetles (Figure 8A).^[^ ^94^ ^]^ The analyte molecules concentrated on the PhC dots due to the wettability difference of hydrophilic and hydrophobic micropatterns and produced strong emission by the fluorescence enhancement effect of the PhC structures. In addition, the PhC chip realized ultrasensitive detection of cocaine molecules after modification with DNA aptamers and reduced the limit of cocaine molecule detection. Furthermore, multi‐component detection also has a strong potential for applications and needs to be improved. Qin et al. reported a rainbow structural‐color chip that enhanced the fluorescence emission over a broad wavelength range to detect different signals in multisaccharide chemical analysis (Figure 8B). Different concentrations of twelve saccharides and their mixtures were successfully distinguished by the structured chip.^[^ ^95^ ^]^ Zhang and co‐workers designed an AIE–doped polyionic liquid‐based PhC sphere detector for differential detection of multiple analytes (Figure 9A).^[^ ^96^ ^]^ One single photonic sphere included the ionic liquid units, which can interact with different analytes for efficient detections. This detector can be responsive to twenty natural amino acids with enhanced selectivity and excellent scalability. The single PhC sphere strategy showed the application potential for precise identification of complex multi‐analyte detection. Zhao's group developed a bioinspired PhC barcode for multiplexed and sensitive nucleic acid detection (Figure 9B).^[^ ^97^ ^]^ Different probes can be immobilized on the surface of PhC spheres with different structural colors to realize multiplex detection. The experiment proved that detecting Human Papilloma Virus nucleic acid by PhC barcodes had ultra‐high sensitivity, high accuracy, and specificity. In summary, these sensors with bio‐inspired structures demonstrated the ability to detect susceptible and multiple substances in biological detections.

### 4.3 Highly bright light sources

**FIGURE 10:**
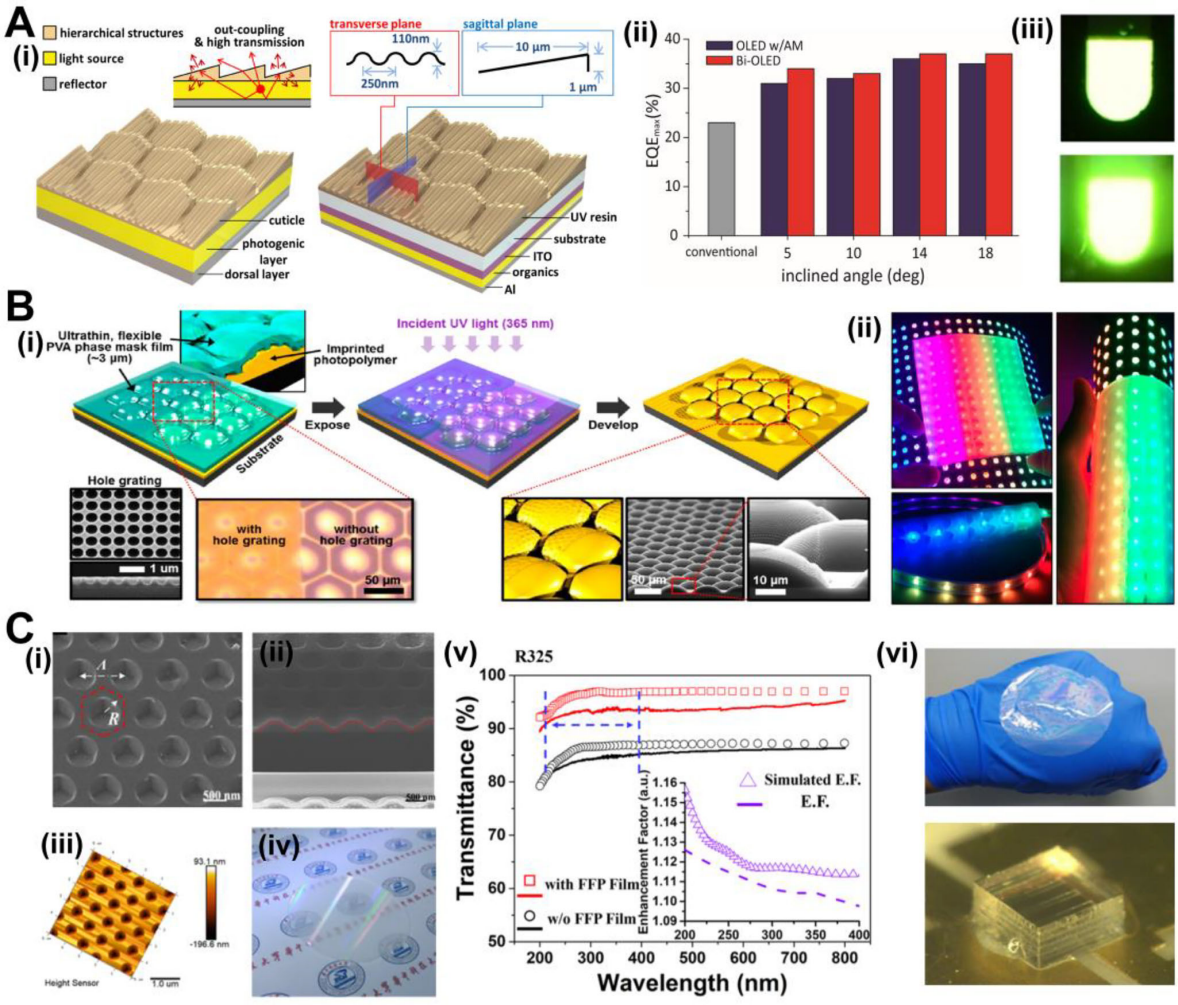
(A) (i) Surface topographies of a firefly lantern template and a bio‐inspired organic OLED. The inset shows the out‐coupling and high transmission effect that happened in a firefly lantern. (ii) Maximum EQE of an OLED device with different structures at four inclined angles. (iii) Photographs of emission from OLEDs with flat (upper) and structured (lower) surfaces. Reproduced with permission.^[^ ^103^ ^]^ Copyright 2016, American Chemical Society. (B) (i) Schematic of fabricating the optical film with moth‐eye‐inspired structures. (ii) Digital photographs of flexible LED array displays covered with large‐area antireflective diffuser films. Reproduced with permission.^[^ ^104^ ^]^ Copyright 2016, American Chemical Society. (C) (i) Top‐view and (ii) cross‐sectional SEM images of the patterned template for nanoimprinting. (iii) The atomic force microscope image and (iv) digital photograph of nanopatterned template. (v) Transmittance of the template with and without the FFP film. The inset plot exhibits the enhancement of effective transmittance. (vi) The photographs of the fabricated FFP film and DUV‐LED with FFP film. Reproduced with permission.^[^ ^105^ ^]^ Copyright 2019, American Chemical Society.

LEDs have developed for many years, and they are now the most commonly used light source. However, the low EQE still needs to be optimized. The EQE is defined as the ratio of photons emitted to the injected electrons. Periodic gratings, microlens arrays, and highly‐order nanorod arrays were used to reduce the optical losses to improve the EQE of LEDs.^[^ ^98^ ^]^ The main optical losses in LED devices include the waveguided modes loss, interface loss between the air and substrate, and plasmon polariton loss. When people face problems about how to reduce the emission losses of light sources, solutions can be found by observing light management phenomena in nature.^[^ ^99^, ^100^, ^101^, ^102^ ^]^ Researchers found many inspirations for enhancing the light extraction of LEDs in the optical structure of living organisms. Kim and co‐workers reproduced a layered structure composed of longitudinal nanostructures with a period of 250 nm and asymmetric microstructures with a width of 10 μm using PDMS by imitating the optical structures in firefly lanterns (Figure 10A).^[^ ^103^ ^]^ This bio‐inspired organic LED (OLED) exhibited enhanced light extraction efficiency by 61% and side‐enhanced Lambertian emission, thus improving the wide‐angle illumination. It is also demonstrated that light extraction efficiency can be increased by introducing external components. Kwon and co‐workers investigated rough‐surface microlens arrays with radius of curvature of ∼28 μm based on moth‐eye surface structures. They fabricated multifunctional optical films with polyvinyl alcohol materials with good physical flexibility and superior conformity (Figure 10B).^[^ ^104^ ^]^ These special films had excellent transparency due to the anti‐reflection effect provided by the moth‐eye nanostructures. Adding this film to LED arrays can exhibit enhanced power efficiency by 4% and reduce power consumption, which is used in flexible and large‐area LED arrays. Liang and co‐workers adopted the nanopatterned imprinting to fabricate the high‐transmittance and flexible fluoropolymer (FFP) films with nanoconical structures for enhancing light extraction efficiency (Figure 10C).^[^ ^105^ ^]^ The TE and transverse magneticmodes of deep ultraviolet (DUV) LEDs can be significantly improved with this approach by 20.5% and 21.8%, respectively. This strategy is cost‐effective from the packaging point of view because it could be scalably fabricated and covered on conventional light‐emitting devices. Park and co‐workers found inspiration from the petal surface of tulips and prepared hierarchical surface structures composed of a wavy surface with a radius of curvature of 50 μm and surface relief grating with a 500 nm period.^[^ ^106^ ^]^ The structures on the films had a special diffraction pattern which helped to improve the outcoupling efficiency of the light‐emitting source.

**FIGURE 11:**
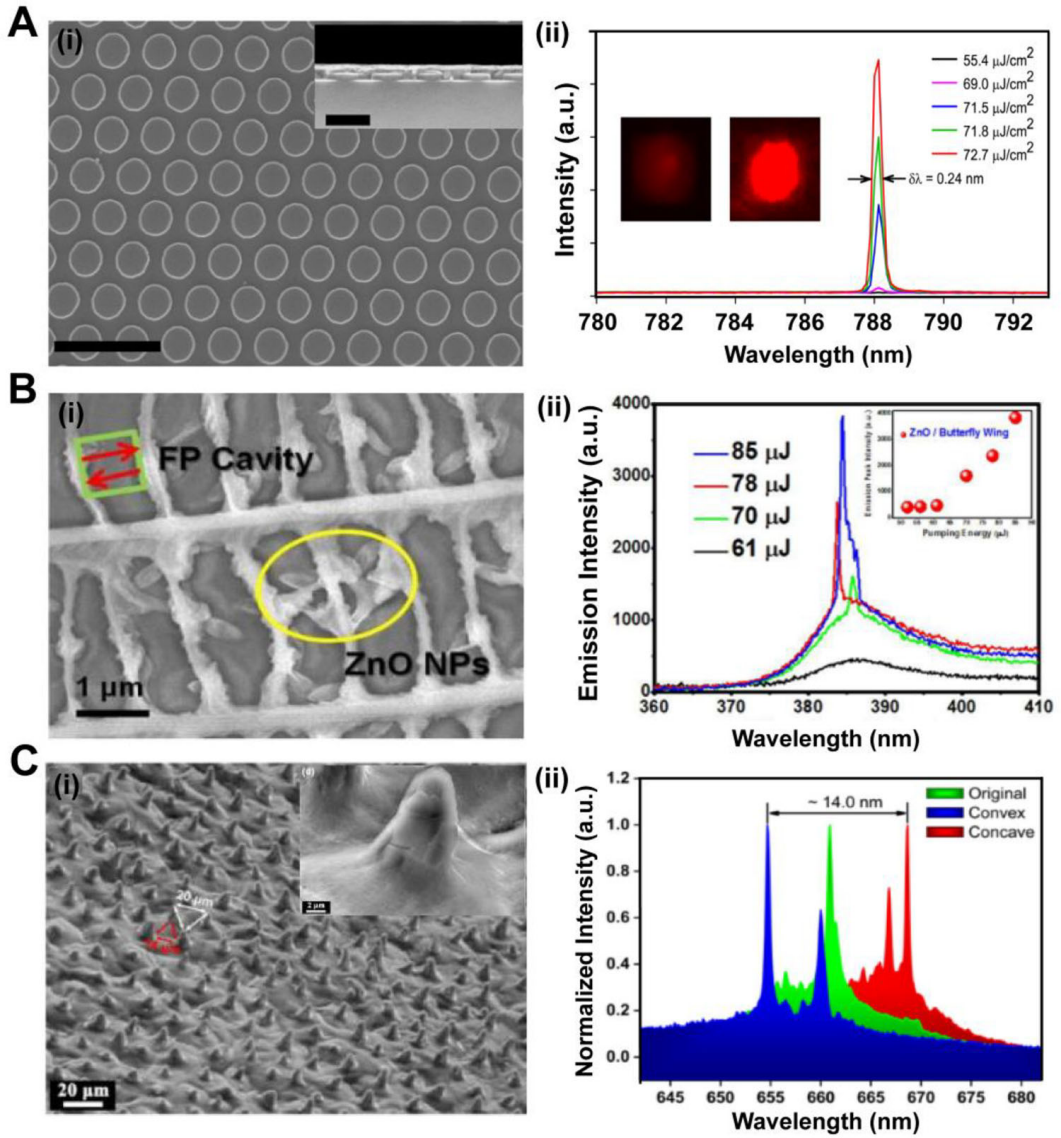
(A) (i) The SEM image of uniformly nanostructured 2D PhC pattern (scale bar is 1 μm), and the inset shows the cross‐sectional SEM image of a patterned sample (scale bar is 400 nm). (ii) Excitation dependence of the lasing spectrum of 2D PhC patterned perovskite film with single‐mode emission. Insets are the IR camera pictures of the patterned film below (left) and above (right) the lasing threshold pump power. Reproduced with permission.^[^ ^108^ ^]^ Copyright 2016, American Chemical Society. (B) (i) The SEM image of wings with the ZnO NPs which formed an F‐P laser. (ii) The intensity spectra of bio‐inspired F‐P laser with increasing pump energy. Inset shows the plot of the lasing threshold test. Reproduced with permission.^[^ ^113^ ^]^ Copyright 2015, Springer Nature. (C) (i) SEM images of the surface structure of bio‐inspired PDMS template. Inset is an SEM image of a single papilla hill structure. (ii) The intensities of random laser in flat (green), convex (blue), and concave (red) states. Reproduced with permission.^[^ ^114^ ^]^ Copyright 2020, American Chemical Society.

Lasers have the characteristics of monochromaticity, directionality, and coherence. They are widely used as light sources in illumination, display, and integrated photonic devices. Optical structures are used to enhance the interaction between photons and materials, leading to improved performance of lasers. For example, PhC structures can serve as microcavities. The strong optical confinements of PhC microcavities can significantly reduce the size of the device and improve the quality factor of the cavity, which is suitable for high‐density and functional integrated optical circuits. Jia and co‐workers fabricated a MAPbI_3_ laser using a one‐dimensional (1D) grating with a 403 nm period as a second‐order distributed feedback structure.^[^ ^107^ ^]^ This continuous wave DFB laser covered with a 50 nm thick gold layer and a 15 nm thick Al_2_O_3_ interlayer can operate for 25 ns with the threshold of 5 kW cm^−2^ under the excitation of InGaN diode laser at 160 K, which provides an idea for the development of electrically pumped perovskite lasers. To further control the modes of lasers, Chen and co‐workers embedded the perovskite thin film into the 2D PhC resonator with a 450 nm period, which formed a single‐mode PhC band‐edge laser with a threshold of 68.5 ± 3.0 μJ cm^−2^ (Figure 11A).^[^ ^108^ ^]^ They realized an arrayed laser display device with this 2D PhC structured laser. Subsequently, Schünemann et al. fabricated perovskite films with an inverse opal structure using 3D PhC as templates.^[^ ^109^ ^]^ A single‐mode distributed feedback laser with stable emission was fabricated by solution process, which did not require expensive and complicated processes. In addition, researchers found that some optical structures in living organisms such as the quasi‐period PhC structures on the wings of butterfly and the random nanostructures on the wings of cicada can also act as resonators for lasing.^[^ ^110^, ^111^, ^112^ ^]^ Wang and co‐workers used butterfly wings with a quasi‐periodic structure as a resonant cavity. They added zinc oxide particles as a gain material and obtained the Fabry–Pérot (F‐P) mode laser emission at 61 μJ pumping energy (Figure 11B).^[^ ^113^ ^]^ Due to the great flexibility of butterfly wings, the output of the laser can be maintained under bending. This work proved that natural biological structures can provide effective optical feedback. Li and co‐workers reproduced the micromastoid structure from the lotus leaf surface by soft lithography (Figure 11C).^[^ ^114^ ^]^ These randomly distributed structures scattered the coherent light and increased the travel time and path of light for enhancing the amplification of lasing. The optical cavity in the shape of an equilateral triangle which consisted of three mastoid structures showed the laser emission. Furthermore, the bio‐inspired laser has tunable emission spectrum by moving the position of excitation or bending the flexible substrate.

## 5 CONCLUSION AND PROSPECT

Light signals in the natural environment are crucial to the evolution and selection of organisms. The biological systems continuously optimize complex tissues and structures to adapt to the changes in the environment. Based on the experiences of luminescence enhancement in biological optical systems, researchers designed bio‐inspired optical structures and fabricated optical devices for information encryption, biochemical detection, and light‐emitting sources. Although many bio‐inspired strategies for enhancing luminescence have been developed in recent years, some organisms and their employed optical phenomena have not yet been clearly explained. Further studies on the properties and functions of optical systems in organisms will be exploited to get optimized bio‐inspired optical morphologies and structures for enhanced luminescence. We hope that the following approaches will address these issues:

For complex applications, the functions and performance of optical structures need to be reconsidered due to different requirements in technical applications. Apart from studying the simple structures and their mechanisms, combining two or more structures also achieve an improved performance of device.^[^ ^115^, ^116^ ^]^ In‐depth study of bio‐inspired structures can be executed through nanoscale characterization and computer simulation techniques.

It is also crucial to enhance the resolution of the bio‐inspired structures by improving the manufacturing processes. Utilizing high‐precision metasurface structures makes it possible to achieve luminescence enhancement in nonlinear optics.^[^ ^117^, ^118^ ^]^ For example, the second and third harmonics are helpful in the research of biological imaging and detection, and they show tremendous application potential.

To solve the need for flexible and wearable devices in the fields of display, lighting, and detection, researchers should continue to develop flexible and large‐area light‐emitting devices, such as flexible displays, wearable biosensors, and smart electronic devices.^[^ ^119^, ^120^, ^121^, ^122^, ^123^, ^124^, ^125^ ^]^ Exploring continuous fabrication technologies is a sensible path to achieving comprehensive coverage of complex optical structures of large‐area devices.^[^ ^126^, ^127^, ^128^ ^]^ The processes also should develop in the direction of simple operation, low costs, and sustainable resources. Reasonable design of materials and structures can avoid the influences of substrates, electrodes, etc., so that the device maintains excellent luminescence characteristics and stability under large strain and geometric deformation. With the development of advanced materials, structures, and processing technologies, enhanced luminescence devices with bio‐inspired optical structures will have bright prospects in information encryption, medical diagnosis, and energy‐saving lighting.

## CONFLICT OF INTEREST STATEMENT

The authors declare no conflicts of interest.
